# Evidence of a Putative Deep Sea Specific Microbiome in Marine Sponges

**DOI:** 10.1371/journal.pone.0091092

**Published:** 2014-03-26

**Authors:** Jonathan Kennedy, Burkhardt Flemer, Stephen A. Jackson, John P. Morrissey, Ferghal O'Gara, Alan D. W. Dobson

**Affiliations:** 1 Marine Biotechnology Centre, Environmental Research Institute, University College Cork, Lee Road, Cork, Ireland; 2 Department of Microbiology, University College Cork, Cork, Ireland; 3 BIOMERIT Research Centre, University College Cork, Cork, Ireland; 4 School of Biomedical Sciences, Curtin University, Perth, Western Australia, Australia; University of Vienna, Austria

## Abstract

The microbiota of four individual deep water sponges, *Lissodendoryx diversichela*, *Poecillastra compressa*, *Inflatella pellicula*, and *Stelletta normani*, together with surrounding seawater were analysed by pyrosequencing of a region of the 16S rRNA gene common to *Bacteria* and *Archaea*. Due to sampling constraints at depths below 700 m duplicate samples were not collected. The microbial communities of *L. diversichela*, *P. compressa* and *I. pellicula* were typical of low microbial abundance (LMA) sponges while *S. normani* had a community more typical of high microbial abundance (HMA) sponges. Analysis of the deep sea sponge microbiota revealed that the three LMA-like sponges shared a set of abundant OTUs that were distinct from those associated with sponges from shallow waters. Comparison of the pyrosequencing data with that from shallow water sponges revealed that the microbial communities of all sponges analysed have similar archaeal populations but that the bacterial populations of the deep sea sponges were distinct. Further analysis of the common and abundant OTUs from the three LMA-like sponges placed them within the groups of ammonia oxidising *Archaea* (*Thaumarchaeota*) and sulphur oxidising *γ-Proteobacteria* (*Chromatiales*). Reads from these two groups made up over 70% of all 16S rRNA genes detected from the three LMA-like sponge samples, providing evidence of a putative common microbial assemblage associated with deep sea LMA sponges.

## Introduction

Marine sponges are important members of marine benthic communities throughout polar, tropical and temperate oceans. They are sessile filter feeding animals, capable of filtering up to 50 thousand litres of seawater per litre of sponge per day [1]. Marine sponges have gained much attention during recent years due to their remarkably dense and diverse community of bacterial, archaeal and eukaryotic microorganisms. This microbiota contributes to sponge biology in many ways, such as providing a chemical defence mechanism, carbon and nitrogen cycling and as a food source [2] [3]. Sponges can also be grouped according to the density of bacteria within their tissues into high microbial abundance (HMA) sponges and low microbial abundance (LMA) sponges [4] with HMA sponges reported to have microbial densities of 10^8^–10^10^ bacteria per g of tissue while LMA sponges have 10^5^–10^6^ bacteria per g of tissue. Great efforts have been made to characterise the diversity of the microbial assemblages in shallow water sponges, and in HMA sponges in particular, using both culture dependent [5]–[8] and culture independent approaches [9]–[11]. Up to 2007, 15 bacterial phyla (including the candidate phylum *Poribacteria*), 2 major archaeal lineages and many microbial eukaryotes had been reported from marine sponges [2]. In recent years the application of “next generation” sequencing has allowed access to the so called “rare biosphere” [12] and increased the number of bacterial phyla detected in sponges to more than 30. Marine sponges from the Great Barrier Reef [3], the Red Sea [13], the Mediterranean [14], the northern Atlantic [15], [16], the Caribbean [17], Brazil [18] and worldwide [19] have been studied for their microbial diversity. More interesting than the sheer diversity of microbial communities which have been found in sponges are the presence of sponge-specific microorganisms, i.e. OTUs found almost exclusively in sponges [20]. In a comprehensive study of sponge-microbe associations, Schmitt *et al.* have distinguished between core, variable and species-specific assemblages in sponges. Interestingly, only a very small proportion of 90%, 95% and 97% OTUs was shared between different sponge species [19]. This “core” community of microbial OTUs found in most studied sponges implies a horizontal transfer of sponge-associated microbial diversity through the surrounding seawater. In previous studies, by comparison of larvae and adult sponges, evidence for vertical symbiont transfer has also been shown [3]. Thus potentially both vertical and horizontal transfer is involved in shaping sponge-associated microbial communities. Other studies have focused on describing the community structures in diverse sponges [3], [15], including archaeal diversity [13], seasonal variations in the community structure [17] and functional analysis of the sponge metagenome [16], [18].

Relative to their shallow water counterparts, little is known about the microbial diversity of deep water sponges, due in no small part to the inherent technical difficulties in obtaining specimens from below 1000 m. Nevertheless, in culture dependent studies Romanenko and co-workers reported the isolation of two new bacterial species from a deep sea sponge [21], [22], while Brück and colleagues identified an *Entotheonella* species in *Discodermia dissoluta* from a depth of 150 m [23] and later characterized the culturable anaerobes from *Geodia* sp. samples from depths of ∼200–350 m [24]. A culture independent study on *Polymastia cf. corticata* sampled at a depth of ∼1100 m revealed that bacteria previously found in shallow water sponges are also present in deep water sponges and that the bacterial community has a spatial distribution in the sponge [25]; a phenomenon which has also been described for a shallow water sponge [26]. Deep water sponges, including a *Lissodendoryx sp.*, [27], [28] have also proven fruitful sources of novel bioactive compounds, many of which are likely to be of microbial origin.

The potential roles of most microorganisms within the sponge microbiota are, as yet, largely unknown. However recent studies using biochemical and metatranscriptomic approaches strongly suggest that ammonia-oxidising *Archaea* (AOA) are actively involved in nitrification within sponges [16], [29]. Roles for other members of the sponge microbiota in processes such as sulphur oxidation and the provision of chemical defence systems are also likely but currently there is little direct evidence of these.

In order to increase our understanding of the microbial communities associated with deep water marine sponges and to assess potential similarities between the microbiota of deep water species, this study has applied the pyrosequencing approach to analyse the microbiome of sponge samples from the bathypelagic zone. Four northern deep water species, *I. pellicula*, *S. normani*, *L. diversichela* and *P. compressa* which have not previously been studied for their associated microbiota, were collected from deep water canyon systems in the North Atlantic at depths of 748, 1350, 1350 and 1469 m respectively. The primer pair targeting the V5–V6 region of 16S rRNA genes which is common between both *Archaea* and *Bacteria* was chosen because *Archaea* have been reported to be particularly abundant in deep water marine sponges [30]. Also, this primer pair yields sequence lengths of about 280 bp which enables the classification of sequence reads into lower taxonomic levels. The results of these analyses have led to the characterisation of the microbial communities associated with these sponge samples and the comparison of these communities has revealed a putative common microbial assemblage associated with deep sea LMA-like sponges. A fuller understanding of the deep sea sponge microbial community is a first step to understanding the roles these microbes play in this as yet poorly understood environment.

## Results

### Sponge sampling

**Figure 1:**
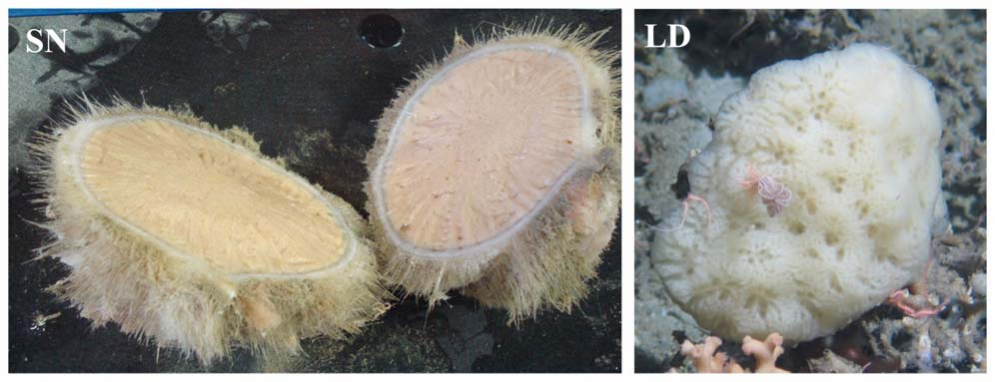
Sponge samples *L. diversichela* (LD) and *S. normani* (SN).

Four sponge samples and water samples were obtained from depths of 700 m to 1500 m (Table 1). Sponge samples (Figure 1) were identified as *L. diversichela* (LD), *S. normani* (SN), *P. compressa* (PC) and *I. pellicula* (IP). Duplicate samples were not available for analyses due to the difficulties associated with sampling at such depths. The cortex and the choanosome of *S. normani* were processed as two separate samples in order to characterize any potential spatial distribution of bacteria in this sponge although for most analyses the data from these samples were combined. The outer layer of the sponge was cleaned carefully with a sterile scalpel in order to remove any sediment attached to the sponge.

**Table 1:** Sponges and water samples and pyrosequence data.

Sample		latitude	longitude	depth [m]	Initial reads	Filtered reads*
***I. pellicula***	IP	54.0015	−12.3100	748	10070	8470
***L. diversichela***	LD	54.0584	−12.5469	1350	6423	6093
***P. compressa***	PC	54.0633	−12.4131	1469	4445	1976
***S. normani***	SN	54.0613	−12.5518	1350	26668	22475
**Seawater**	W-1	54.0015	−12.3100	748	6207	5863
**Seawater**	W-2	54.0630	−12.4165	1500	18328	17044
**Seawater**	W-3	54.0584	−12.5469	1350	9103	8661

The sponge and water samples were collected at depths from 700 m to 1500 m in canyon regions north of Porcupine Bank in the N. Atlantic.

*After applying Chimera Slayer from reads grouped into OTUs at 97% similarity.

### Taxonomic richness

A total number of 81,244 individual 16S rRNA sequence reads were obtained by pyrosequencing. Errors in the dataset were analysed and corrected using Acacia [31]. The average sequence length of all quality filtered sequences was ∼280 bp. Following these quality checks a total of 70,582 sequences were analysed (Table 1) using QIIME [32]. Operational taxonomic units (OTUs) were selected at 97%, 94%, and 90% similarities and potentially chimeric sequences were removed using ChimeraSlayer following alignment. A total of 6,357 (97% similarity) OTUs were obtained from the sponge and water samples with 4,504 OTUs from the sponge samples alone (Table 2). The sample from sponge *Stelleta normani* had the most diverse population with 3,942 97% OTUs detected in this single sponge sample. The other sponge samples had OTUs ranging from 172 to 247, while OTUs in the water samples ranged from 382 to 1,213.

**Table 2:** Diversity of sponge and water samples.

Sample	97%			94%			90%		
	OTUs	Chao1	Shannon	OTUs	Chao1	Shannon	OTUs	Chao1	Shannon
*I. pellicula*	172	374	2.27	139	293	2.14	108	212	2.09
*L. diversichela*	247	506	1.43	205	350	1.31	159	236	1.27
*P. compressa*	178	741	2.98	143	428	2.75	116	221	2.75
*S. normani*	3942	9750	8.50	1945	3002	7.28	593	704	6.19
Water-1	611	1036	6.88	456	677	6.48	285	394	5.64
Water-2	1213	2346	6.42	879	1633	5.92	517	794	5.49
Water-3	382	700	3.79	320	498	3.58	232	332	3.30

Number of OTUs and species richness estimates of the seawater and sponge-associated microbial communities. Chao1 and Shannon indices were determined at 97, 94 and 90% similarities of 16 s rRNA sequences.

The rank abundance curves at 97% sequence similarity (Figure S1) show that the microbial communities of the samples from sponges *L. diversichela* and *I. pellicula* are dominated by a relatively small number of OTUs, demonstrated through the steep slope. The communities of the other samples are more evenly distributed with *S. normani* having the most even distribution. The rarefaction curve (Figure S2) of the microbial associates of the *S. normani* sample does not reach a plateau, indicating that even with over 22,000 reads there is an undersampling of the microbial biodiversity. The rarefaction curves of the other samples indicated that a greater proportion of the biodiversity was sampled although two of the water samples also indicated that there was some under sampling of these.

### Bacterial vs. archaeal diversity

The relative abundance of archaeal reads in the sponge samples ranged from 4% in LD, 19% in SN, 48% in IP and up to 65% in PC, while in the accompanying surrounding seawater samples the *Archaea* made up 36–38% of all reads. *Archaea* were more often found in the cortex of the *S. normani* sample (28% relative abundance) than in the choanosome (10%). In all samples, bacterial reads made up the remaining proportion with a negligible amount of sequencing reads not classified into either of the two prokaryotic domains of life.

### Archaeal diversity

**Figure 2:**
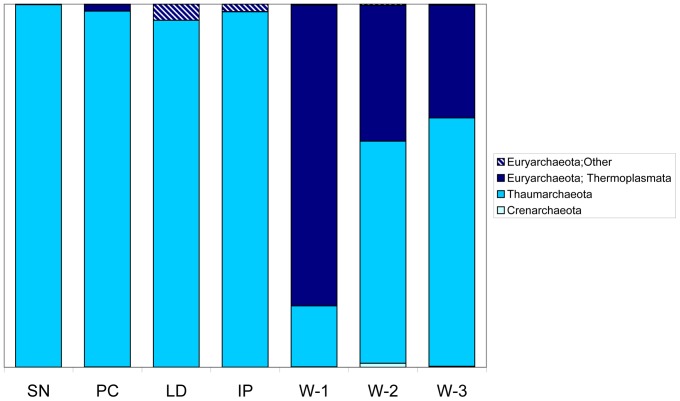
Archaeal diversity in sponge and seawater samples. OTUs were grouped at 97% similarity and taxonomy assigned using the RDP classifier with the Greengenes database of assigned sequences. Refer to Table 1 for sample abbreviation.

In all sponge and water samples the two archaeal phyla *Euryarchaeota* and *Thaumarchaeota* were present (Figure 2). *Crenarchaeota* were detected at very low levels (two single reads) in two of the samples (W2 and W3). A total of 192 (97%) OTUs were classified as *Archaea*. Among the sponge samples there were 61 (97%) OTUs within the *Archaea* with the *Thaumarchaeota* being the most diverse *Archaea* present (46 OTUs). Over 95% of all archaeal reads in the sponge samples were *Thaumarchaeota* with the remainder classified as *Euryarchaeota*. The seawater samples contained both *Thaumarchaeota* and *Euryarchaeota* with the *Thaumarchaeota* fraction consisting of 17% (W-1), 62% (W-2) and 68% (W-3) of archaeal reads. Of the *Thaumarchaeota* fraction the vast majority of the seawater-derived reads and those from sponge IP were classified in the candidate genus *Nitrosopumilus*, whereas reads from the remaining sponges were mainly classified in the family *Cenarchaeaceae*. In each sponge sample over 70% of the *Thaumarchaeota* fraction was made up of up to three (97%) OTUs. Among reads classified as *Euryarchaeota* there were no abundant OTUs from the sponge samples, with the water samples each containing several OTUs in the marine group II and III *Thermoplasmata*.

**Figure 3:**
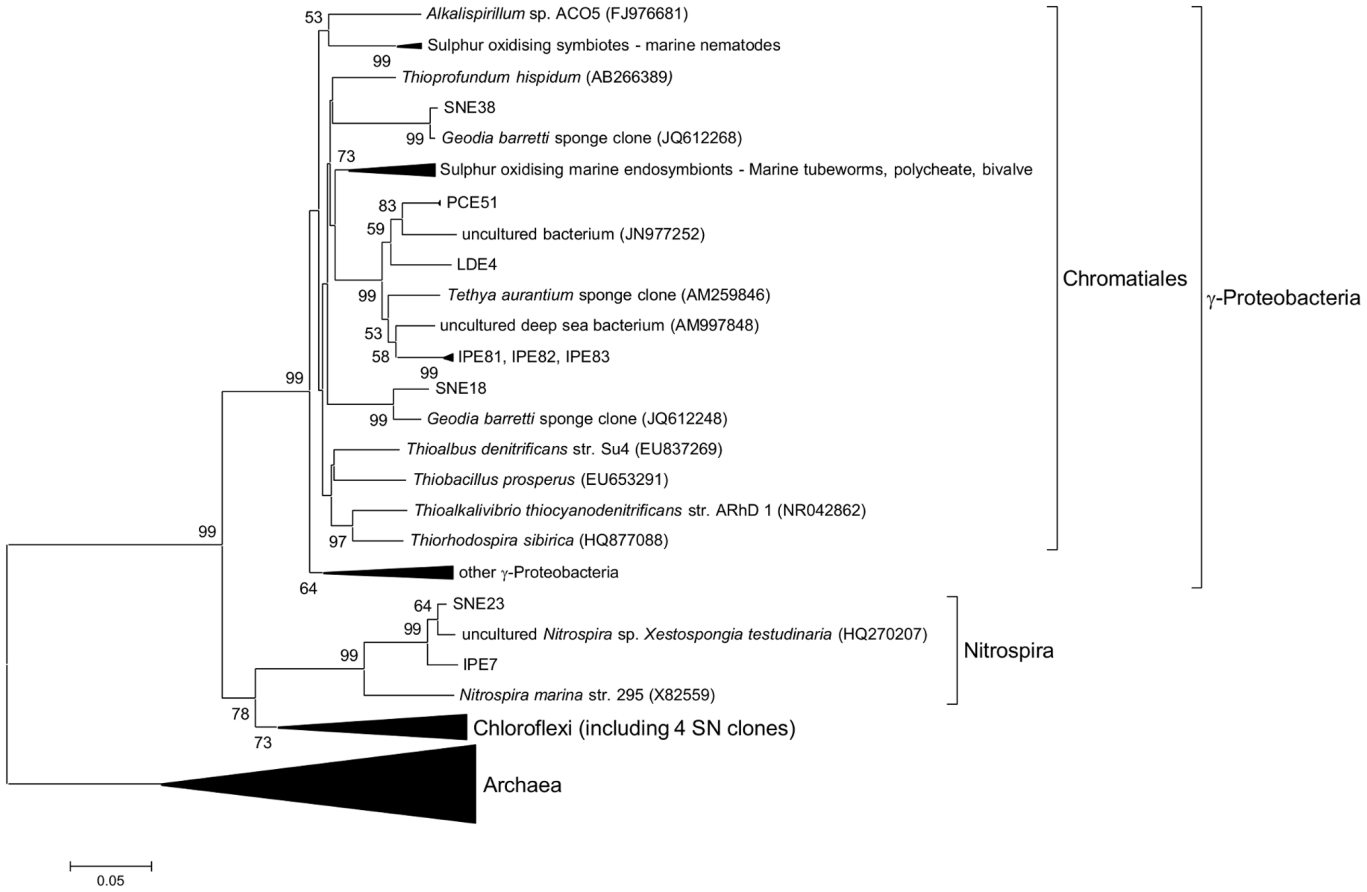
Phylogenetic relationship of archaeal 16S rRNA clones from deep sea sponges. 16S rRNA sequences were determined from cloned PCR amplicons (also see Table 3). Sequences were aligned using PyNast and neighbour joining phylogenetic tree was constructed using MEGA5. Bootstrap values (500 replicates) greater than 50% are shown next to the branches.

To allow further analysis of the archaeal members of the sponge associated communities, longer fragments of the archaeal 16S rRNA genes were amplified and cloned. Each of these sponge libraries was found to contain clones that were close matches to abundant amplicons from the respective pyrosequencing libraries. All the archaeal 16S rRNA gene sequences recovered from the clone libraries were analysed by BLAST analysis and by taxonomic classification with Greengenes, RDP and Silva pipelines. Phylogenetic analysis (Figure 3) and Greengenes taxonomy (Table 3) showed that all sponge archaeal clones grouped with the Marine Group I *Thaumarchaeota*. Phylogenetic analysis revealed two distinct clades; a clade of sponge-derived reads from sponges *I. pellicula*, *P. compressa* and *S. normani* that clustered together with *Nitrosopumilus maritimus* and the sponge symbiont *Cenarchaeum symbiosum*; and a clade consisting of clones from sponges *L. diversichela* and *P. compressa* that clustered with seawater-derived archaeal reads (Figure 3).

**Table 3:** Greengenes classification of 16S rRNA clones from deep sea sponges.

Clone(s)	Greengenes Simrank id	Greengenes DNAML id	Greengenes taxonomy					
			Kingdom	Phylum	Class	Order	Family	Genus/Species
LDA22, LDA24, PCA21	83–92	0.96–0.99	*Archaea*	*Crenarchaeota*	*Thaumarchaeota*	*Cenarchaeales*	*Cenarchaeaceae*	*Nitrosopumilus*
LDA19, LDA23, LDA25, LDA26, PCA22	87–94	0.96–0.99	*Archaea*	*Crenarchaeota*	*Thaumarchaeota*	*Cenarchaeales*	*Cenarchaeaceae*	*Nitrosopumilus*
PCA18, PCA19	73–76	0.95–0.96	*Archaea*	*Crenarchaeota*	*Thaumarchaeota*	*Cenarchaeales*	*Cenarchaeaceae*	*Cenarchaeum symbiosum*
IPA1	91	0.98	*Archaea*	*Crenarchaeota*	*Thaumarchaeota*	*Cenarchaeales*	*Cenarchaeaceae*	*Nitrosopumilus*
SNA18	72	0.94	*Archaea*	*Crenarchaeota*	*Thaumarchaeota*	*Cenarchaeales*	*Cenarchaeaceae*	
SNE31, SNE32, SNE35, SNE36	49–76	0.89–0.96	*Bacteria*	*Chloroflexi*	*Chloroflexi-4*			
SNE23,,IPE7	80–84	0.97–0.99	*Bacteria*	*Nitrospirae*	*Nitrospira*	*Nitrospirales*	*Nitrospiraceae*	*Nitrospira*
PCE51, LDE4, SNE18, SNE38, IPE13	61–82	0.93–0.98	*Bacteria*	*Proteobacteria*	*γ-Proteobacteria*	*Chromatiales*		
IPE82	55	0.90	*Bacteria*	*Proteobacteria*	*γ-Proteobacteria*	*Chromatiales*	*Ectothiorhodospiraceae*	*Thiohalospira halophila*
IPE81	55	0.90	*Bacteria*	*Proteobacteria*	*γ-Proteobacteria*	*Chromatiales*	*Ectothiorhodospiraceae*	*Thiorhodospira*
IPE83	54	0.90	*Bacteria*	*Proteobacteria*	*γ-Proteobacteria*			endosymbiont of *Siboglinum fiordicum*
PCE4, SNE27	45–76	0.89–0.96	*Bacteria*	*Actinobacteria*	*Actinobacteria*	*Acidimicrobiales*		
LDE5	95	0.99	*Bacteria*	*Proteobacteria*	*δ-Proteobacteria*	*Desulfobacterales*	*Nitrospinaceaea*	*Nitrospina*
SNE21, SNE26	64–84	0.94–0.98	*Bacteria*	*Gemmatimonadetes*	*Gemmatimonadetes*			
SNE29	74	0.94	*Bacteria*	*Acidobacteria*	*PAUC37f*			
SNE25	67	94	*Bacteria*	*Acidobacteria*	*Solibacteres*	*Solibacterales*	*Solibacteraceaea*	*Solibacter*
SNE28	48	0.84	*Bacteria*	*Bacteroidetes*	*Sphingobacteria*	*Sphingobacteriales*	*Flammeovirgaceaea*	

16S rRNA sequences of archaeal and bacterial origin were amplified. The DNA sequence of cloned amplicons was determined and analysed using the Greengenes classifier.

### Bacterial diversity

**Figure 4:**
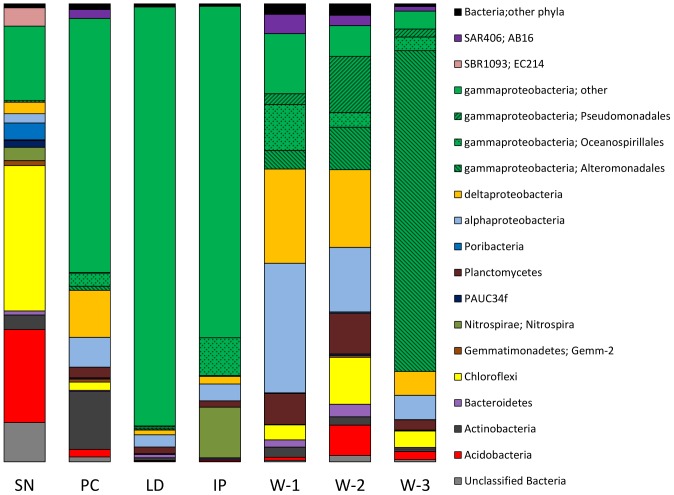
Bacterial diversity in sponge and seawater samples. OTUs were grouped at 97% similarity and taxonomy assigned using the RDP classifier with the Greengenes database of assigned sequences. Refer to Table 1 for sample abbreviation.

A total of 6,160 bacterial OTUs were detected at 97% similarity with 4,506 bacterial OTUs (97%) present within the sponge reads (Figure 4). These OTUs were assigned to a total of 35 bacterial phyla (including candidate phyla) with 28 phyla detected in the sponge-derived reads. The most diverse phylum of bacteria within the sponge samples were the *Chloroflexi* with 1,487 individual OTUs (97%) present (this was reduced to 118 OTUs when sequences were grouped at 90% sequence similarity), the *Proteobacteria* were the next most diverse phylum with 722 OTUs (97%). The proteobacterial OTUs could be further subdivided into 317 *γ-Proteobacteria*, 128 *δ-Proteobacteria* and 167 *α-Proteobacteria*. Other diverse groups of bacteria present included the *Acidobacteria* with 321 OTUs (97%), the *Poribacteria* with 114 OTUs (97%), the *Actinobacteria* with 110 OTUs (97%), *Gemmatimonadetes* with 54 OTUs (97%) and *Bacteroidetes* with 48 OTUs (97%). A large number of 97% OTUs (1297) were classified only at the domain level but these were conflated to 234 when a 90% similarity cut off was used. A summary of OTU numbers at different similarity groupings is shown in Table 2.

A number of candidate divisions/Phyla were also detected in addition to the *Poribacteria*; GN02, OP3, SAR406, TM7, and ZB3 were all detected with low read abundance and diversity, with candidate divisions/Phyla SBR1093 and WS3 being more diverse (23 OTUs (97%) and 25 OTUs (97%) respectively) in the sponge samples.

### Analysis at lower taxonomic levels

Analysis at lower taxonomic levels revealed that the *Thaumarchaea* present in all sponges were classified in the genera *Cenarchaeum* and *Nitrosopumilus* (Figure 3). From genomic analysis of the sponge symbiont *Cenarchaeum symbiosum* and physiological studies of cultured *Nitrosopumilus* these species are known to belong to the group of ammonia-oxidising archaea (AOA) capable of oxidising ammonia to nitrite [33]–[35]. Other microorganisms present within the sponges with potential contribution to nitrogen cycling are the nitrite oxidising bacteria *Nitrospiraceae*, *Nitrospina* and the ammonia oxidising bacteria *Nitrosomonadales*. Reads classified to *Nitrospira* were found in three of the sponge samples though they were more prevalent in SN and IP, whereas *Nitrospina* classified reads were found in all sponge samples.

Of the other common phyla detected within the sponges *Acidobacteria* group 6, and *Chloroflexi* SAR202 were found in all sponge samples and were especially common in SN (Figure 3). These groups are typical members of the sponge microbiota and have been found in numerous studies of sponges from diverse habitats including sponges from shallow, tropical environments [13] and from deeper, cold water environments [16]. However all sponge samples contained OTUs consisting of large numbers of reads that were classified only at the level of the class *γ-Proteobacteria*. When the data was analysed at 90% similarity grouping, many of these OTUs grouped together to form single OTUs.

**Figure 5:**
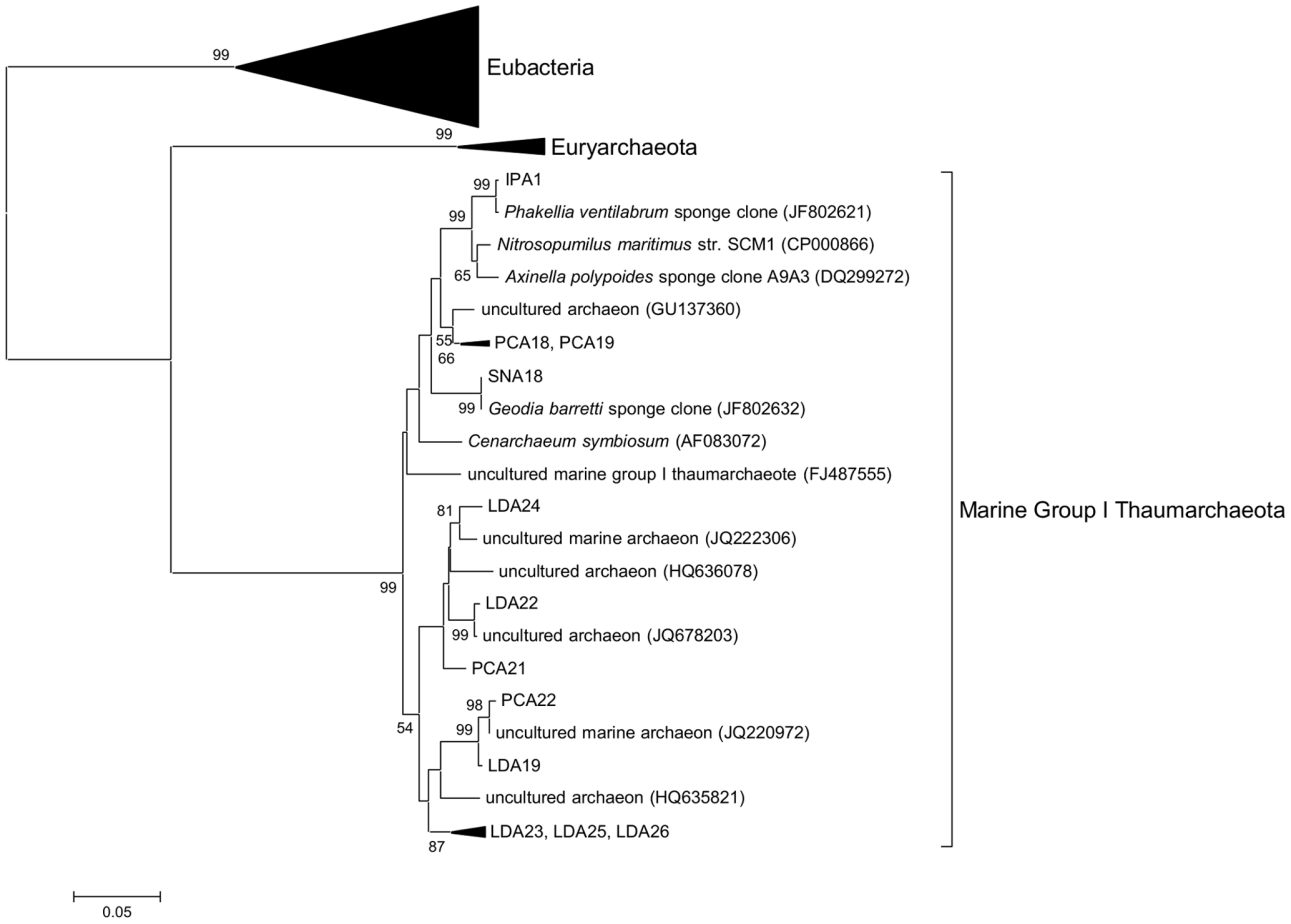
Phylogenetic relationship of bacterial 16S rRNA clones from deep sea sponges. 16S rRNA sequences were determined from cloned PCR amplicons (also see Table 3). Sequences were aligned using PyNast and neighbour joining phylogenetic tree was constructed using MEGA5. One representative sequence per OTU was used for alignment and construction of tree. Bootstrap values (500 replicates) greater than 50% are shown next to the branches.

As these unclassified γ-proteobacterial reads appeared to represent an abundant OTU within the sponges we sought to study these in greater detail. To allow for more robust phylogenetic analyses than are possible with the shorter reads generated by pyrosequencing near full length 16S rRNA libraries were prepared from each sponge metagenome with several clones (4–30) sequenced from each. Comparison to the pyrosequencing data showed that each of these small libraries contained representative clones of the most common OTUs detected in the pyrosequencing data (including unclassified *γ-Proteobacteria*, *Nitrospira*, *Chloroflexi* and others). These sequences were analysed further by BLAST analysis, by taxonomic assignment using RDP, Greengenes and Silva pipelines and by alignment and phylogenetic analyses. Greengenes taxonomic assignment classified the previously unclassified γ-proteobacterial OTUs within the *Chromatiales* or with sulphur oxidising symbionts (Table 3). Likewise phylogenetic analysis of these clones showed that all these 16S rRNA sequences were found to cluster within the *Chromatiales* group of *γ-Proteobacteria* together with other sponge-derived sequences and endosymbionts from tubeworms (Figure 5). All cultured members of these groups and characterised symbionts are believed to be involved in sulphide oxidation [36]–[38]. Other sequenced clones confirmed the presence of multiple *Chloroflexi* from *S. normani*, *Nitrospira* and *Nitrospina* bacteria together with other groups detected from the pyrosequencing analysis (*Acidimicrobiales*, *Gemmatimonadetes* and *Acidobacteria*).

### Analyses of sponge communities

Robust comparison of sponge species specific microbial communities was not possible due to the lack of duplicate sponge samples, however shared features of the deep sea sponge microbial communities were examined. In order to determine whether there was a shared deep-sea sponge microbial community the compositions of the sponge microbial communities were analysed by direct comparison of shared OTUs. Classified reads were first grouped into OTUs at 97%, 94% and 90% sequence similarity, OTUs shared between sponge samples were then analysed to determine if there was a common microbiota. For the purposes of this study the shared microbiota was defined as OTUs that were present in at least 3 of the 4 sponge species analysed (Table 4).

**Table 4:** Analysis of OTUs shared between deep sea sponge samples.

OTU similarity grouping	*S. normani*		*L. diversichela*		*I. pellicula*		*P. compressa*	
	Shared OTUs	% of reads	Shared OTUs	% of reads	Shared OTUs	% of reads	Shared OTUs	% of reads
97%	2	0.02%	4	3%	3	0.1%	3	1.5%
94%	13	4%	22	93%	20	86%	20	71%
90%	24	25%	36	95%	37	86%	32	87%

OTUs were determined at 97, 94 and 90% similarities. OTUs were considered to be shared if present in three of the four sponges. The proportion of the total reads made up of the shared OTUs is indicated.

**Figure 6:**
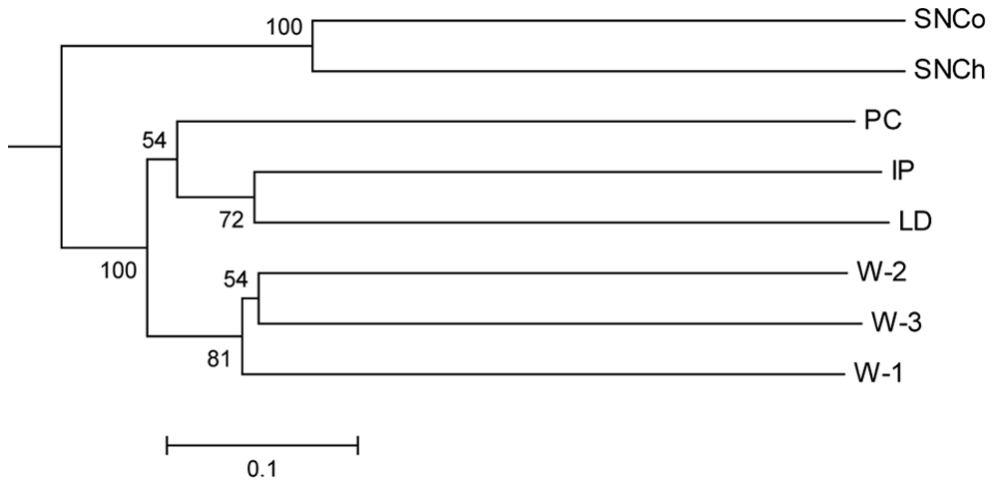
Relationship between microbial communities of deep sea sponges and seawater by UniFrac UPGMA clustering. See table 1 for sample abbreviations. The pyrosequencing data for the *Stelletta normani* cortex (SNCo) and choanosome (SNCh) samples were analysed separately. Jackknife values are shown at nodes. The scale bar indicates the Unifrac distance.

At all sequence similarity groupings (90%, 94% and 97%) over 70% of all OTUs were present within just a single sponge sample. At 97% similarity grouping there were very few shared OTUs with only 3 OTUs shared between 3 or more sponge samples, and 25 present in 2 or more. The analysis of shared OTUs at 94% similarity gave 23 OTUs that were present in 3 or more sponge samples and at 90% similarity there were 37 OTUs present in 3 or more sponge samples (Table 4). While the number of OTUs shared between the sponge samples was low compared to the overall number of OTUs present, when the number of reads associated with the shared OTUs were analysed it was found that the common microbiota (at 90% OTU similarity) comprised 25% (SN), 86% (IP), 87% (PC), and 95% (LD) of the total number of analysed reads for each sponge sample. The microbiota of the sponge and seawater samples was also compared using UniFrac UPGMA cluster analysis. The relationship between the samples shows that the three water samples from the different depths and locations have similar microbial communities and form a cluster (Figure 6). The sponge samples clustered into two groups, one consisting of the choanosome and cortex from the two samples of SN and the other cluster consisting of the microbiota of IP, PC and LD. Thus the microbial communities of the two tissue types from the *S. normani* sample are highly similar as are the communities from the single samples of sponges *I. pellicula*, *P. compressa*, and *L. diversichela* (Figure 6).

The shared microbiota was found to contain 3 OTUs (90%) classified as *Thaumarchaeota* (also in Figure 3) comprising a total of over 95% of all reads characterised as *Archaea*. In addition the shared microbiota was found to contain an OTU within the group of *γ-Proteobacteria* described above as grouping with the *Chromatiales*. This OTU comprised 69%, 41% and 90% of the bacterial reads from IP, PC and LD respectively. A related OTU within the *Chromatiales* group (identified by BLAST and phylogenetic analysis) from SN comprised 6% of the bacterial reads from this sponge sample (Figure 5). While the common sponge community described above accounted for over 75% of all reads in sponge samples from IP, PC, and LD, the SN sponge sample had a more complex community with the presence of many reads classified as *Acidobacteria*, *Poribacteria*, *Chloroflexi*, *Nitrospira* and candidate SBR1093 (Figure 3). The high diversity and overall makeup of the microbial community of the SN sample is similar to that found in high microbial abundance (HMA) sponges from other marine environments [39] and this data clearly supports the classification of SN as a deep water HMA sponge. In contrast, the dominance of IP, PC and LD by a few OTUs is typical of low microbial abundance (LMA) sponges [40] and these three sponge samples are likely to be examples of deep sea LMA sponges.

### Comparison to other sponge communities

To compare the microbial communities of the deep sea sponges that were the subject of this study with other sponge microbial communities the dataset from a study of sponges collected from the Red Sea at depths of 8–19 m was analysed [13]. These data were generated using the same primer pairs as used in this study so a direct comparison of the data was possible. The Red Sea sponge data were reanalysed together with the deep sea data.

Comparison of shared OTUs grouped at 90% similarity indicated that there were similarities in the archaeal communities (Table 5). One archaeal OTU, classified as *C. symbiosum* was present in all samples making up a large proportion (20–98%) of all reads classified as *Archaea*, while two other OTUs classified as *Archaea* were detected in 6 of the 7 sponge samples. These three common archaeal OTUs together accounted for 65–99% of total archaeal reads (Table 5A). One bacterial OTU, classified as *Acidobacteria-6*, was found in all samples, however the abundance of these reads was low (<0.1%) in three of the sponge samples. Five other bacterial OTUs were found in 6 of the 7 sponge samples, however all these shared bacterial OTUs made up only 1–7% of the total reads (Table 5A). In contrast, analysis of the Red Sea shallow water sponges revealed 103 shared OTUs in total, 6 archaeal OTUs and 97 bacterial OTUs, together these shared OTUs constituted 82–93% of the archaeal reads and 63–79% of the bacterial reads from the Red Sea shallow water sponges indicating a high degree of similarity in the microbial communities of these sponges (Table 5B). Of the 97 bacterial OTUs common to the Red Sea shallow water sponges, only 7–11 were present in the deep sea LMA sponges and these made up 1–23% of the total bacterial reads (Table 5B). A total of 27 OTUs were found to be common to the deep sea LMA sponges, 5 archaeal and 22 bacterial. These shared bacterial OTUs made up a total of 67–94% of the deep sea bacterial reads (Table 5C). Of the bacterial OTUs common to the deep sea LMA sponges, 3–14 were also detected in the Red Sea shallow water sponges, however these were found to make up only a small component (2–5%) of the bacterial population of the Red Sea sponges, highlighting differences in the microbial community structures.

**Table 5:** Analysis of OTUs shared between deep sea sponges and Red Sea sponges.

			Red Sea Sponges						Deep Sea Sponges							
			*X. testudinaria*		*H. erectus*		*S. carteri*		*S. normani*		*L. diversichela*		*P. compressa*		*I. pellicula*	
			(HMA)		(HMA)		(LMA)		(HMA)		(LMA)		(LMA)		(LMA)	
			OTUs	% of reads	OTUs	% of reads	OTUs	% of reads	OTUs	% of reads	OTUs	% of reads	OTUs	% of reads	OTUs	% of reads
A	Shared OTUs:	Archaea	3	65%	2	74%	2	68%	3	98%	3	94%	3	97%	3	99%
	All sponges	Bacteria	5	4%	5	3%	6	5%	5	7%	6	1%	5	6%	5	2%
B	Shared OTUs:	Archaea	6	93%	6	91%	6	82%	1	91%	1	78%	1	97%	1	90%
	Red Sea sponges	Bacteria	97	76%	97	63%	97	79%	34	45%	7	1%	8	6%	11	23%
C	Shared OTUs:	Archaea	3	65%	2	74%	3	68%	4	98%	5	99%	5	98%	5	99%
	deep sea LMA sponges	Bacteria	3	3%	7	2%	14	5%	6	6%	22	94%	22	67%	22	73%

OTUs were determined at 90% similarity. For Row A OTUs were considered shared between all sponges if present in 6 of the 7 sponges. For Rows B and C OTUs were considered shared if present in all 3 of the Red Sea shallow water sponges or deep sea LMA samples respectively. Figures in the table indicate the total number of OTUs shared in each class for each sponge and the % of archaeal or bacterial reads these OTUs encompassed.

**Figure 7:**
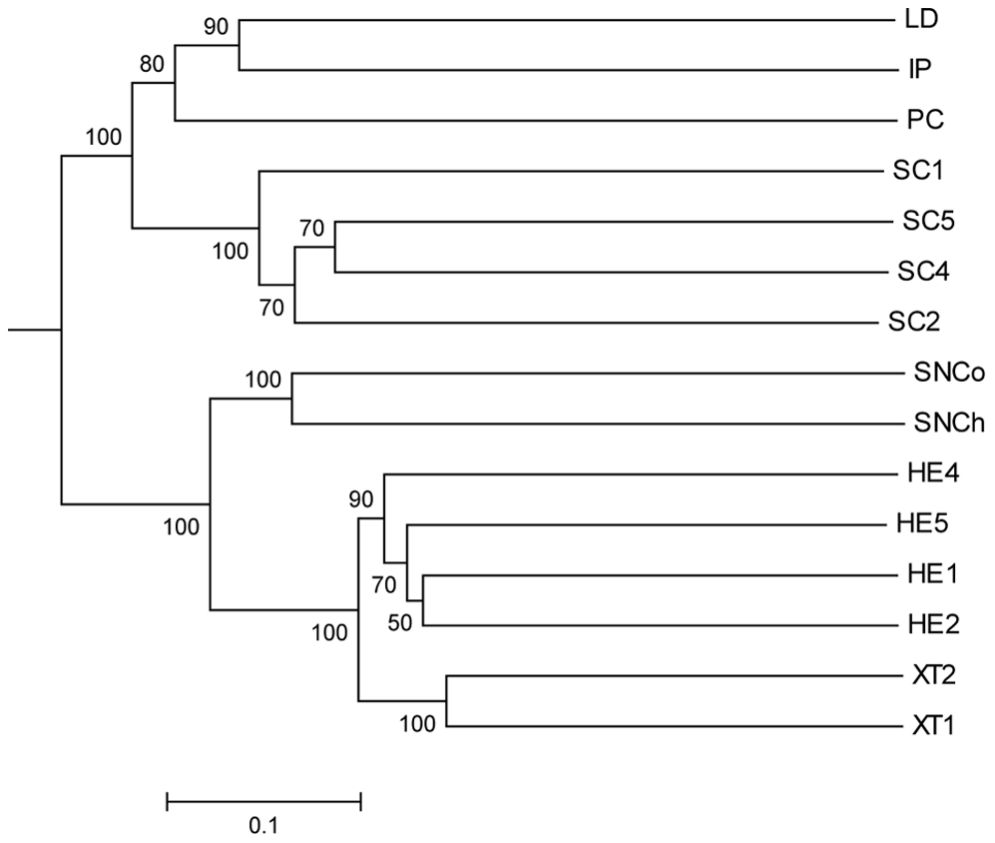
Relationship between microbial communities of deep sea sponges and Red Sea sponges by UniFrac UPGMA clustering. See Table 1 and Figure 6 for sample abbreviations for deep sea sponges. The pyrosequencing data for the *Stelletta normani* cortex (SNCo) and choanosome (SNCh) samples were analysed separately. Red Sea sponge samples SC-*Stylissa carteri*, HE-*Hyrtios erectus*, XT-*Xestospongia testudinaria*. Jackknife values are shown at nodes. The scale bar indicates the Unifrac distance.

The microbial communities of deep sea and Red Sea shallow water sponges were also analysed using UniFrac UPGMA cluster analysis (Figure 7). The results of this largely supported the analysis of the shared OTUs, the microbiota of the three LMA-type deep water sponges (LD, IP and PC) were found to be more similar to each other than to other sponge or water samples with the microbiota of the LMA Red Sea sponge *Stylissa carteri* being more closely related than other sponge or water samples. The microbiota of SN was found to be more distinct but again was more related to the microbiota of the Red Sea sponges *Xestospongia testudinaria* (a confirmed HMA sponge) and *Hyrtios erectus* (a presumed HMA sponge). The UniFrac analysis thus supports the classification of the sponges LD, IP and PC as LMA and SN as HMA.

## Discussion

Marine sponges have long been known to harbour a wide range of microbes and while the microbial community of shallow water sponges has been studied extensively, relatively little is known about the microbes associated with deep water sponges, especially from the bathypelagic and deeper zones. By applying the 454 pyrosequencing approach to assess the microbial communities of deep-water sponge samples this study sheds further light on microbes associated with these sponges.

### Bacterial community

The lack of duplicate sponge samples in this study means that robust conclusions about the microbial communities associated with individual deep seawater sponge species are not feasible. Howewer the shared microbiota of these deep sea sponge species has been analysed to determine whether there is a specific microbial community associated with deep water sponges. The analysis of shared OTUs between the deep sea sponge samples revealed that most of the OTUs present were unique to individual sponge samples, however there was also a number of OTUs that were shared between at least 3 of the 4 deep water sponge species investigated, suggesting a shared core community in these deep water sponges. Although sequence abundance can not be considered truly quantitative due to biases in PCR amplification and differences in 16S rRNA gene copy number these OTUs were found, in some cases, to constitute very large proportions of the total reads in the study, indicating that they are likely to make up substantial parts of the sponge microbial communities. Analysis of pyrosequencing read abundance has proved insightful in a recent study describing and demonstrating differences in microbial-host specificity in LMA sponges [41]. Microbes within these abundant groups included the AOA *Thaumarchaeota*, a group which contains the sponge symbiont *C. symbiosum*. A second group of microbes present in all sponge samples were identified as *γ-Proteobacteria* within the *Chromatiales* group. In three of the sponge samples IP, LD and PC these two microbial groupings made up over 70% of all reads. This lower diversity microbial community is typical of low-microbial abundance (LMA) sponges, however other studies of LMA sponges have concluded that each sponge species harbours a unique bacterial community [40], while the data here suggest a potential common microbiota for deep sea LMA sponges. The data from this study also confirm other recent reports that LMA sponges have microbial communities that are distinct from and are not merely a reflection of surrounding seawater [40], [41]. Direct comparison of pyrosequencing data, including shallow water LMA sponges, with this dataset revealed that the microbial communities of all the sponges have similar archaeal populations but that the bacterial populations of the deep sea sponges are distinct. Other non-pyrosequencing based studies of LMA sponges collected from shallow temperate and tropical waters show that LMA sponges typically have a single large bacterial OTU that can be cyanobacterial or proteobacterial, and that appears to be species specific [40]. This is in contrast to the deep sea LMA sponges which, from the data presented here, appear to have a more conserved bacterial population structure. Two additional studies of deep sea sponge microbiota provide additional support for this; analysis of the microbial community of a single sample of a deep sea *Polymastia* sponge by DGGE analysis of 16S rRNA genes and *aprA* (adenylyl-sulphate reductase) genes revealed the presence of sulphur-oxidising *γ-Proteobacteria* (*Chromatiales*) and marine group I *Thaumarchaeota* [25]. Likewise in another study bacterial 16S rRNA genes amplified from two individual deep sea sponge samples were analysed by DGGE and the dominant bands were shown to be derived from thioautotrophic *γ-Proteobacteria* [37]. These studies, together with the deeper pyrosequencing data presented here provide additional evidence for a common microbial community structure in deep sea sponges.

AOA have previously been shown to be transcriptionally and metabolically active in nitrification in cold water sponges [16], [29] and nitrification has also been detected in LMA sponges [42], thus it seems likely that the AOA groups detected in these sponges may have similar metabolic capabilities. The other abundant group, thioautotrophic *γ-Proteobacteria* has previously been found within sponges [11], [37], and associated with other marine invertebrates such as marine tubeworms and shellfish [38]. This class of bacteria appears to be a frequent symbiont, present in many marine invertebrates. Analysis of the seawater derived sequences revealed that at the 90% similarity threshold these *γ-Proteobacteria* were essentially absent from seawater with a single read from one seawater sample grouping into the shared OTU. While it is possible that these bacteria are acquired from seawater they are clearly concentrated within the sponge tissue and appear to dominate the microbial communities of several sponge species. The SN sponge sample has a microbiota more typical of high-microbial abundance (HMA) sponges, with many bacterial species present and typical phyla such as *Nitrospira*, *Acidobacteria*, *Poribacteria* and *Chloroflexi* being observed. However this sponge also appears to have some similarities with the other deep water sponges under study; namely the presence of a common *Thaumarchaeota* OTU and a *Chromatiales* OTU, which together make up 19% of all reads in SN.

### Evidence of a deep sea specific sponge community?

The role of these *Archaea* and *Bacteria* within deep sea sponges is unknown, however the presence of two groups of microorganism whose closest studied relatives are chemolithoautotrophic is striking. The *Thaumarchaeota* are AOA, gaining energy through oxidation of ammonia while the sulphur oxidising *γ-Proteobacteria* gain energy through oxidation of sulphide and other sulphur compounds. As previously mentioned, AOA have been shown to be major contributors to nitrification in cold water sponges and in the sponge *Geodia barretti* archaeal transcripts predicted to be involved in ammonia oxidation are highly abundant [16]. A symbiotic relationship between marine tubeworms and sulphur oxidising bacteria is well established, with tubeworms dependent on nutrients supplied by sulphur oxidising chemoautotrophic *γ-Proteobacteria* [43]. A similar role for sulphur oxidising bacteria within sponges has not been shown although these bacteria appear to be widespread and abundant in deep sea sponges [37], [38]. There is also a report of a specific putative symbiosis between free-living marine *Thaumarchaeota* and sulphur-oxidising *γ-Proteobacteria* in sulphide rich mangrove swamps [44], implying that these two organisms themselves can form a close symbiotic relationship. How these common chemolithoautotrophic microorganisms contribute to the physiology of deep sea sponges is as yet unknown but their consistent presence implies a potentially important symbiotic relationship.

## Conclusion

This study presents an analysis of the shared prokaryotic diversity of four individual deep-water sponges and shows that the microbiota of these deep-water sponges share features with their shallow water counterparts. All sponge samples were found to contain diverse *Bacteria* and *Archaea*, and among the *Archaea* present a group classified as *C. symbiosum* was present in all samples. Three of the individual sponges in the study *L. diversichela*, *I. pellicula* and *P. compressa* were classified as LMA sponges on the basis of their microbial communities. The microbial composition of the *S. normani* sample includes many OTUs from groups commonly found in HMA marine sponges. Pyrosequencing data indicated that the LMA sponges *L. diversichela*, *I. pellicula* and *P. compressa* were dominated by two microbial groups; AOA *C. symbiosum* group and sulphur-oxidising *γ-Proteobacteria*, providing evidence of a putative common deep sea LMA sponge microbial community consisting of ammonia oxidising *Archaea* and sulphur oxidising *γ-Proteobacteria*. The data presented here are derived from four individual sponge samples and additional data will be required to determine if this apparent deep sea LMA sponge microbial community is shared with other deep sea sponges. Due to the nature of the current study the contribution of these common and apparently abundant members of the deep-sea sponge community to the shared metabolism of the sponge community is unknown but it is likely that these abundant microbes play an important role in sponge biology.

## Methods

### Sample collection

Specific permission was not required, to obtain the marine sponge samples used in this study as they were collected in Irish territorial water, by an Irish research vessel, funded by the Irish government. The sponge samples do not involve endangered or protected sponge species. The sponge samples used in this study were collected with the remotely operated vehicle (R.O.V.) *Holland I* during the Biodiscovery cruise 2010 aboard R.V. *Celtic Explorer*. Upon retrieval the sponge samples were washed with sterile, artificial seawater (33.3 g/L Instant Ocean, Aquarium Systems – Blacksburg, VA, USA) and stored at −80°C until molecular work was carried out in our laboratories in Cork. A part of each sample was also used for taxonomic identification by Christine Morrow, Queens University, Belfast. Additionally, a water sample was retrieved during a CTD (Conductivity-Temperature-Depth) measurement. 30 L water were collected as close as possible to the sponge sampling site, filtered through a 0.2 µm membrane filter (Whatman – Austin, TX, USA) and the filter was immediately frozen at −80°C. Depths and GPS location of samples are indicated in Table 1. Duplicate samples were not collected due to sampling difficulties at such depths.

### Metagenomic DNA extraction from seawater and sponge samples

DNA was extracted from filters using WaterMaster DNA Purification Kit (Epicentre Biotechnologies, Madison, WI, USA) according to the manufacturer's instructions. Extracted DNA was stored at −20°C.

The sponge tissue (3–5 g) was cut into fine pieces with a sterile razorblade and then ground to a fine powder under liquid nitrogen using a mortar and pestle. For the *S. normani* sample, the cortex was first separated from the choanosome, cleaned carefully with sterile artificial seawater and any remaining sediment from the surface was removed with a sterile razor blade. For the choanosome a cross section of the ball shaped tissue was taken in order to include inner- and outer areas of the choanosome. While processed separately reads from choanosome and cortex were ultimately pooled for some analyses. The ground sponge tissue was added to a lysis buffer adapted from Brady (100 mM Tris, 100 mM EDTA, 1.5 M NaCl (w/v), 1% CTAB (w/v), 2% SDS (w/v); 5 ml buffer per 1 g sponge tissue; [45]) and incubated for 2 h at 70°C. Metagenomic DNA was then extracted as previously described [11]. DNA solutions were analysed by gel electrophoresis, quantified by spectrophotometry (NanoDrop ND-1000-Wilmington, DE, USA) and then stored at −20°C.

### PCR amplihcon library preparation for pyrosequencing

PCR amplicon libraries of the V5–V6 region of 16S rRNA genes were prepared from all metagenomic DNAs. Universal primers U789f (5′-TAGATACCCSSGTAGTCC-3′) and U1068r (5′-CTGACGRCRGCCATGC-3′), targeting both bacteria and archaea [13], were adapted for pyrosequencing by the addition of sequencing adapters and multiplex identifier (MID) sequences as per Table S1. Each 50 µl PCR reaction comprised 1× buffer, 0.2 mM dNTPs (both Fermentas, St. Leon-Rot, Germany), 0.1 µM of each primer (Sigma Aldrich, Arklow, Ireland), 2 U Taq polymerase (Fermentas), ∼10 ng template DNA and dH_2_O. PCR cycle conditions were as reported previously [13]. To minimise PCR bias three individual reactions were performed per template and equimolar amounts of PCR products from each of the three reactions were pooled for pyrosequencing. PCR products were purified using Qiagen PCR Purification Kit (Qiagen Ltd., UK) as per the manufacturer's instructions. Barcoded samples were pooled and sequenced on a GS FLX Titanium platform (454 Life Sciences) at the University of Liverpool, Centre for Genomic Research, Liverpool, UK.

### Pyrosequencing data analysis

Initial quality filtering and barcode assignment of reads was performed using the QIIME package [32]. Sequences shorter than 200 nucleotides, with an average quality score <25, or with >6 ambiguous bases were removed from the analysis. All reads were then analysed for errors and corrected using Acacia1.52 [31]. Reads were then grouped into OTUs at 97%, 94% and 90% sequence similarities using uclust. Each OTU was then assigned to a taxonomic group using the RDP classifier at 50% confidence and the Greengenes database of assigned sequences [46]. OTUs were then aligned to the pre-aligned Greengenes 16S data using PyNAST [47]. Potentially chimeric sequences were identified and removed from the dataset using ChimeraSlayer [48].

Rarefaction and rank abundance curves were calculated from OTU tables generated at 97% similarity using alpha diversity and rank abundance scripts within the QIIME pipeline. Shannon indices and Chao1 species estimators were calculated from OTU tables generated at 90%, 94% and 97% similarity using alpha diversity scripts within the QIIME pipeline.

Raw sequences are deposited in MG-RAST [49] and the NCBI short read archive with the following MG-RAST ID and BioSample numbers; IP – 4533583.3 SAMN02402455; LD – 4533578.3, SAMN02402456; PC – 4533577.3, SAMN02402457; SNCh – 4533579.3, SAMN02402458; SNCo – 4533580.3, SAMN02402459; W-1 – 4533582.3, SAMN02402460, W-2 – 4533576.3, SAMN02402461; W-3 – 4533581.3, SAMN02402462.

### Cloning and analyses of near full length 16S rRNA genes

To generate longer sequences, suitable for more detailed phylogenetic analyses 16S rRNA genes were amplified from sponge metagenomic DNAs using primer sets 27f/1492r for eubacterial 16S rRNA genes [50] and Arch21F/Arch958R for archaeal 16S rRNA genes [51]. PCR amplicons were cloned into pJET1.2 (Thermo Scientific) and a selection of clones from each library were sequenced. Each of these libraries was found to contain sequences that were near-identical (>99%) to the most abundant reads in the V5–V6 libraries. Sequences were trimmed and aligned using PyNast [47] and analysed for chimaeras using ChimeraSlayer [48]. Phylogenetic trees were then calculated in MEGA5 [52] using Neighbour-joining [53] and Maximum likelihood [54] algorithms. Near full length 16S rRNA sequences of clones from this study have been deposited at GenBank with accession numbers KF597097–KF597136.

## Supporting Information

Figure S1 Diversity of microbial communities in deep sea sponges and seawater. Rank abundance curve based on OTUs at 97% similarity. See Table 1 for sample abbreviations.
(TIF)

Figure S2 Diversity of microbial communities in deep sea sponges and seawater. Rarefaction curve based on OTUs at 97% similarity. See Table 1 for sample abbreviations.
(TIF)

Table S1 Primer design including Multiplex Identifier (MID).
(DOC)

## References

[1] Weisz JB, Lindquist N, Martens CS (2008) Do associated microbial abundances impact marine demosponge pumping rates and tissue densities. Oecologia 155: 367–376 10.1007/s00442-007-0910-0 18030495

[2] Taylor MW, Radax R, Steger D, Wagner M (2007) Sponge-associated microorganisms: evolution, ecology, and biotechnological potential. Microbiol Mol Biol Rev 71: 295–347 10.1128/MMBR.00040-06 17554047 PMC1899876

[3] Webster NS, Taylor MW, Behnam F, Lücker S, Rattei T, et al (2010) Deep sequencing reveals exceptional diversity and modes of transmission for bacterial sponge symbionts. Environ Microbiol 12: 2070–2082 10.1111/j.1462-2920.2009.02065.x 21966903 PMC2936111

[4] Hentschel U, Fieseler L, Wehrl M, Gernert C, Steinert M, et al.. (2003) Microbial diversity of marine sponges. In: Mueller W, editor. Marine Molecular Biotechnology. Berlin: Springer. pp. 59–88. 10.1007/978-3-642-55519-0_3 15825640

[5] Hentschel U, Schmid M, Wagner M, Fieseler L, Gernert C, et al (2001) Isolation and phylogenetic analysis of bacteria with antimicrobial activities from the Mediterranean sponges *Aplysina aerophoba* and *Aplysina cavernicola* . FEMS Microbiol Ecol 35: 305–312. 11311441 10.1111/j.1574-6941.2001.tb00816.x

[6] Webster NS, Hill RT (2001) The culturable microbial community of the Great Barrier Reef sponge *Rhopaloeides odorabile* is dominated by an *α-Proteobacterium* . Marine Biology 138: 843–851 10.1007/s002270000503

[7] Kennedy J, Baker P, Piper C, Cotter PD, Walsh M, et al (2009) Isolation and analysis of bacteria with antimicrobial activities from the marine sponge *Haliclona simulan*s collected from Irish waters. Mar Biotechnol (NY) 11: 384–396 10.1007/s10126-008-9154-1 18953608

[8] Flemer B, Kennedy J, Margassery LM, Morrissey JP, O'Gara F, et al (2012) Diversity and antimicrobial activities of microbes from two Irish marine sponges, *Suberites carnosus* and *Leucosolenia* sp. J Appl Microbiol 112: 289–301 10.1111/j.1365-2672.2011.05211.x 22129274

[9] Webster NS, Watts JE, Hill RT (2001) Detection and phylogenetic analysis of novel crenarchaeote and euryarchaeote 16S ribosomal RNA gene sequences from a Great Barrier Reef sponge. Mar Biotechnol (NY) 3: 600–608 10.1007/s10126-001-0065-7 14961332

[10] Thiel V, Neulinger SC, Staufenberger T, Schmaljohann R, Imhoff JF (2007) Spatial distribution of sponge-associated bacteria in the Mediterranean sponge *Tethya aurantium* . FEMS Microbiol Ecol 59: 47–63 10.1111/j.1574-6941.2006.00217.x 17059482

[11] Kennedy J, Codling CE, Jones B V, Dobson ADW, Marchesi JR (2008) Diversity of microbes associated with the marine sponge, *Haliclona simulans*, isolated from Irish waters and identification of polyketide synthase genes from the sponge metagenome. Environ Microbiol 10: 1888–1902 10.1111/j.1462-2920.2008.01614.x 18430018

[12] Sogin ML, Morrison HG, Huber JA, Mark Welch D, Huse SM, et al (2006) Microbial diversity in the deep sea and the underexplored “rare biosphere”. Proc Natl Acad Sci U S A 103: 12115–12120 10.1073/pnas.0605127103 16880384 PMC1524930

[13] Lee OO, Wang Y, Yang J, Lafi FF, Al-Suwailem A, et al (2011) Pyrosequencing reveals highly diverse and species-specific microbial communities in sponges from the Red Sea. ISME J 5: 650–664 10.1038/ismej.2010.165 21085196 PMC3105750

[14] Schmitt S, Hentschel U, Taylor MW (2011) Deep sequencing reveals diversity and community structure of complex microbiota in five Mediterranean sponges. Hydrobiologia 687: 341–351 10.1007/s10750-011-0799-9

[15] Jackson SA, Kennedy J, Morrissey JP, O'Gara F, Dobson ADW (2012) Pyrosequencing reveals diverse and distinct sponge-specific microbial communities in sponges from a single geographical location in Irish waters. Microb Ecol 64: 105–116 10.1007/s00248-011-0002-x 22281804

[16] Radax R, Rattei T, Lanzen A, Bayer C, Rapp HT, et al (2012) Metatranscriptomics of the marine sponge *Geodia barretti*: tackling phylogeny and function of its microbial community. Environ Microbiol 14: 1308–1324 10.1111/j.1462-2920.2012.02714.x 22364353

[17] White JR, Patel J, Ottesen A, Arce G, Blackwelder P, et al (2012) Pyrosequencing of bacterial symbionts within *Axinella corrugata* sponges: diversity and seasonal variability. PLoS One 7: e38204 10.1371/journal.pone.0038204 22701613 PMC3373494

[18] Trindade-Silva AE, Rua C, Silva GGZ, Dutilh BE, Moreira APB, et al (2012) Taxonomic and functional microbial signatures of the endemic marine sponge *Arenosclera brasiliensis* . PLoS One 7: e39905 10.1371/journal.pone.0039905 22768320 PMC3388064

[19] Schmitt S, Tsai P, Bell J, Fromont J, Ilan M, et al (2012) Assessing the complex sponge microbiota: core, variable and species-specific bacterial communities in marine sponges. ISME J 6: 564–576 10.1038/ismej.2011.116 21993395 PMC3280146

[20] Simister RL, Deines P, Botté ES, Webster NS, Taylor MW (2012) Sponge-specific clusters revisited: a comprehensive phylogeny of sponge-associated microorganisms. Environ Microbiol 14: 517–524 10.1111/j.1462-2920.2011.02664.x 22151434

[21] Romanenko LA, Uchino M, Tanaka N, Frolova GM, Mikhailov V V (2008) *Lysobacter spongiicola* sp. nov., isolated from a deep-sea sponge. Int J Syst Evol Microbiol 58: 370–374 10.1099/ijs.0.65391-0 18218933

[22] Romanenko LA, Uchino M, Falsen E, Frolova GM, Zhukova N V, et al (2005) *Pseudomonas pachastrellae* sp. nov., isolated from a marine sponge. Int J Syst Evol Microbiol 55: 919–924 10.1099/ijs.0.63176-0 15774686

[23] Brück WM, Sennett SH, Pomponi SA, Willenz P, McCarthy PJ (2008) Identification of the bacterial symbiont *Entotheonella* sp. in the mesohyl of the marine sponge *Discodermia* sp. ISME J 2: 335–339 10.1038/ismej.2007.91 18256706

[24] Brück WM, Brück TB, Self WT, Reed JK, Nitecki SS, et al (2010) Comparison of the anaerobic microbiota of deep-water *Geodia* spp. and sandy sediments in the Straits of Florida. ISME J 4: 686–699 10.1038/ismej.2009.149 20090787

[25] Meyer B, Kuever J (2008) Phylogenetic diversity and spatial distribution of the microbial community associated with the Caribbean deep-water sponge *Polymastia cf. corticata* by 16S rRNA, *apr*A, and *amo*A gene analysis. Microb Ecol 56: 306–321 10.1007/s00248-007-9348-5 18193317 PMC2755779

[26] Thiel V, Leininger S, Schmaljohann R, Brümmer F, Imhoff JF (2007) Sponge-specific bacterial associations of the Mediterranean sponge *Chondrilla nucula* (*Demospongiae, Tetractinomorpha*). Microb Ecol 54: 101–111 10.1007/s00248-006-9177-y 17364249

[27] Gunasekera SP, Zuleta IA, Longley RE, Wright AE, Pomponi SA (2003) Discorhabdins S, T, and U, new cytotoxic pyrroloiminoquinones from a deep-water Caribbean sponge of the genus *Batzella* . J Nat Prod 66: 1615–1617 10.1021/np030292s 14695808

[28] Hickford SJH, Blunt JW, Munro MHG (2009) Antitumour polyether macrolides: four new halichondrins from the New Zealand deep-water marine sponge *Lissodendoryx* sp. Bioorg Med Chem 17: 2199–2203 10.1016/j.bmc.2008.10.093 19081259

[29] Radax R, Hoffmann F, Rapp HT, Leininger S, Schleper C (2012) Ammonia-oxidizing archaea as main drivers of nitrification in cold-water sponges. Environ Microbiol 14: 909–923 10.1111/j.1462-2920.2011.02661.x 22176665

[30] Pape T, Hoffmann F, Quéric N-V, Juterzenka K, Reitner J, et al (2006) Dense populations of Archaea associated with the demosponge *Tentorium semisuberites* Schmidt, 1870 from Arctic deep-waters. Polar Biology 29: 662–667 10.1007/s00300-005-0103-4

[31] Bragg L, Stone G, Imelfort M, Hugenholtz P, Tyson GW (2012) Fast, accurate error-correction of amplicon pyrosequences using Acacia. Nat Methods 9: 425–426 10.1038/nmeth.1990 22543370

[32] Caporaso JG, Kuczynski J, Stombaugh J, Bittinger K, Bushman FD, et al (2010) QIIME allows analysis of high-throughput community sequencing data. Nat Methods 7: 335–336 10.1038/nmeth.f.303 20383131 PMC3156573

[33] Preston CM, Wu KY, Molinski TF, DeLong EF (1996) A psychrophilic crenarchaeon inhabits a marine sponge: *Cenarchaeum symbiosum* gen. nov., sp. nov. Proc Natl Acad Sci U S A 93: 6241–6246. 8692799 10.1073/pnas.93.13.6241 PMC39006

[34] Hallam SJ, Konstantinidis KT, Putnam N, Schleper C, Watanabe Y, et al (2006) Genomic analysis of the uncultivated marine crenarchaeote *Cenarchaeum symbiosum* . Proc Natl Acad Sci U S A 103: 18296–18301 10.1073/pnas.0608549103 17114289 PMC1643844

[35] Walker CB, de la Torre JR, Klotz MG, Urakawa H, Pinel N, et al (2010) *Nitrosopumilus maritimus* genome reveals unique mechanisms for nitrification and autotrophy in globally distributed marine crenarchaea. Proc Natl Acad Sci U S A 107: 8818–8823 10.1073/pnas.0913533107 20421470 PMC2889351

[36] Kubota N, Kanemori M, Sasayama Y, Masato A, Fukumori Y (2007) Identification of Endosymbionts in *Oligobrachia mashikoi* (*Siboglinidae, Annelida*). Microbes and Environments 22: 136–144.

[37] Nishijima M, Lindsay DJ, Hata J, Nakamura A, Kasai H, et al (2010) Association of thioautotrophic bacteria with deep-sea sponges. Mar Biotechnol (NY) 12: 253–260 10.1007/s10126-009-9253-7 20221658 PMC2891489

[38] Arellano SM, Lee OO, Lafi FF, Yang J, Wang Y, et al (2013) Deep sequencing of *Myxilla* (*Ectyomyxilla*) *methanophila*, an epibiotic sponge on cold-seep tubeworms, reveals methylotrophic, thiotrophic, and putative hydrocarbon-degrading microbial associations. Microb Ecol 65: 450–461 10.1007/s00248-012-0130-y 23052927

[39] Schmitt S, Deines P, Behnam F, Wagner M, Taylor MW (2011) *Chloroflexi* bacteria are more diverse, abundant, and similar in high than in low microbial abundance sponges. FEMS Microbiol Ecol 78: 497–510 10.1111/j.1574-6941.2011.01179.x 22066885

[40] Giles EC, Kamke J, Moitinho-Silva L, Taylor MW, Hentschel U, et al (2013) Bacterial community profiles in low microbial abundance sponges. FEMS Microbiol Ecol 83: 232–241 10.1111/j.1574-6941.2012.01467.x 22882238

[41] Moitinho-Silva L, Bayer K, Cannistraci C V, Giles EC, Ryu T, et al (2013) Specificity and transcriptional activity of microbiota associated with low and high microbial abundance sponges from the Red Sea. Mol Ecol 10.1111/mec.12365 23957633

[42] Schläppy M-L, Schöttner SI, Lavik G, Kuypers MMM, Beer D, et al (2009) Evidence of nitrification and denitrification in high and low microbial abundance sponges. Marine Biology 157: 593–602 10.1007/s00227-009-1344-5 24391241 PMC3873014

[43] Raggi L, Schubotz F, Hinrichs K-U, Dubilier N, Petersen JM (2013) Bacterial symbionts of *Bathymodiolus* mussels and *Escarpia* tubeworms from Chapopote, an asphalt seep in the southern Gulf of Mexico. Environ Microbiol 15: 1969–1987 10.1111/1462-2920.12051 23279012

[44] Muller F, Brissac T, Le Bris N, Felbeck H, Gros O (2010) First description of giant *Archaea* (*Thaumarchaeota*) associated with putative bacterial ectosymbionts in a sulfidic marine habitat. Environ Microbiol 12: 2371–2383 10.1111/j.1462-2920.2010.02309.x 21966926

[45] Brady SF (2007) Construction of soil environmental DNA cosmid libraries and screening for clones that produce biologically active small molecules. Nat Protoc 2: 1297–1305 10.1038/nprot.2007.195 17546026

[46] DeSantis TZ, Hugenholtz P, Larsen N, Rojas M, Brodie EL, et al (2006) Greengenes, a chimera-checked 16S rRNA gene database and workbench compatible with ARB. Appl Environ Microbiol 72: 5069–5072 10.1128/AEM.03006-05 16820507 PMC1489311

[47] Caporaso JG, Bittinger K, Bushman FD, DeSantis TZ, Andersen GL, et al (2010) PyNAST: a flexible tool for aligning sequences to a template alignment. Bioinformatics 26: 266–267 10.1093/bioinformatics/btp636 19914921 PMC2804299

[48] Haas BJ, Gevers D, Earl AM, Feldgarden M, Ward D V, et al (2011) Chimeric 16S rRNA sequence formation and detection in Sanger and 454-pyrosequenced PCR amplicons. Genome Res 21: 494–504 10.1101/gr.112730.110 21212162 PMC3044863

[49] Meyer F, Paarmann D, D'Souza M, Olson R, Glass EM, et al (2008) The metagenomics RAST server - a public resource for the automatic phylogenetic and functional analysis of metagenomes. BMC Bioinformatics 9: 386 10.1186/1471-2105-9-386 18803844 PMC2563014

[50] Lane D (1991) 16S/23S rRNA Sequencing. In: Stackebrandt E, Goodfellow M, editors. Nucleic Acids Techniques in Bacterial Systematics. Chichester: John Wiley & Sons. pp. 115–138.

[51] DeLong EF (1992) *Archaea* in Coastal Marine Environments. Proc Natl Acad Sci U S A 89: 5685–5689 10.1073/pnas.89.12.5685 1608980 PMC49357

[52] Tamura K, Peterson D, Peterson N, Stecher G, Nei M, et al (2011) MEGA5: molecular evolutionary genetics analysis using maximum likelihood, evolutionary distance, and maximum parsimony methods. Mol Biol Evol 28: 2731–2739 10.1093/molbev/msr121 21546353 PMC3203626

[53] Tamura K, Nei M, Kumar S (2004) Prospects for inferring very large phylogenies by using the neighbor-joining method. Proc Natl Acad Sci U S A 101: 11030–11035 10.1073/pnas.0404206101 15258291 PMC491989

[54] Tamura K, Nei M (1993) Estimation of the number of nucleotide substitutions in the control region of mitochondrial DNA in humans and chimpanzees. Mol Biol Evol 10: 512–526. 8336541 10.1093/oxfordjournals.molbev.a040023

