# Correction: Evidence of a Putative Deep Sea Specific Microbiome in Marine Sponges

**DOI:** 10.1371/journal.pone.0103400

**Published:** 2014-07-18

**Authors:** The PLOS ONE Staff

The fifth author's name is spelled incorrectly. The correct name is: Fergal O'Gara. The publisher apologizes for this error.

## References

[1] Kennedy J, Flemer B, Jackson SA, Morrissey JP, O'Gara F, et al (2014) Evidence of a Putative Deep Sea Specific Microbiome in Marine Sponges. PLoS ONE 9(3): e91092 doi:10.1371/journal.pone.0091092 24670421 10.1371/journal.pone.0091092 PMC3966782

