# Supplementary figures and images for: Evidence of a Putative Deep Sea Specific Microbiome in Marine Sponges
Source: PLoS One. 2014 Mar 26;9(3):e91092. doi: 10.1371/journal.pone.0091092 (PMC3966782; doi:10.1371/journal.pone.0091092)

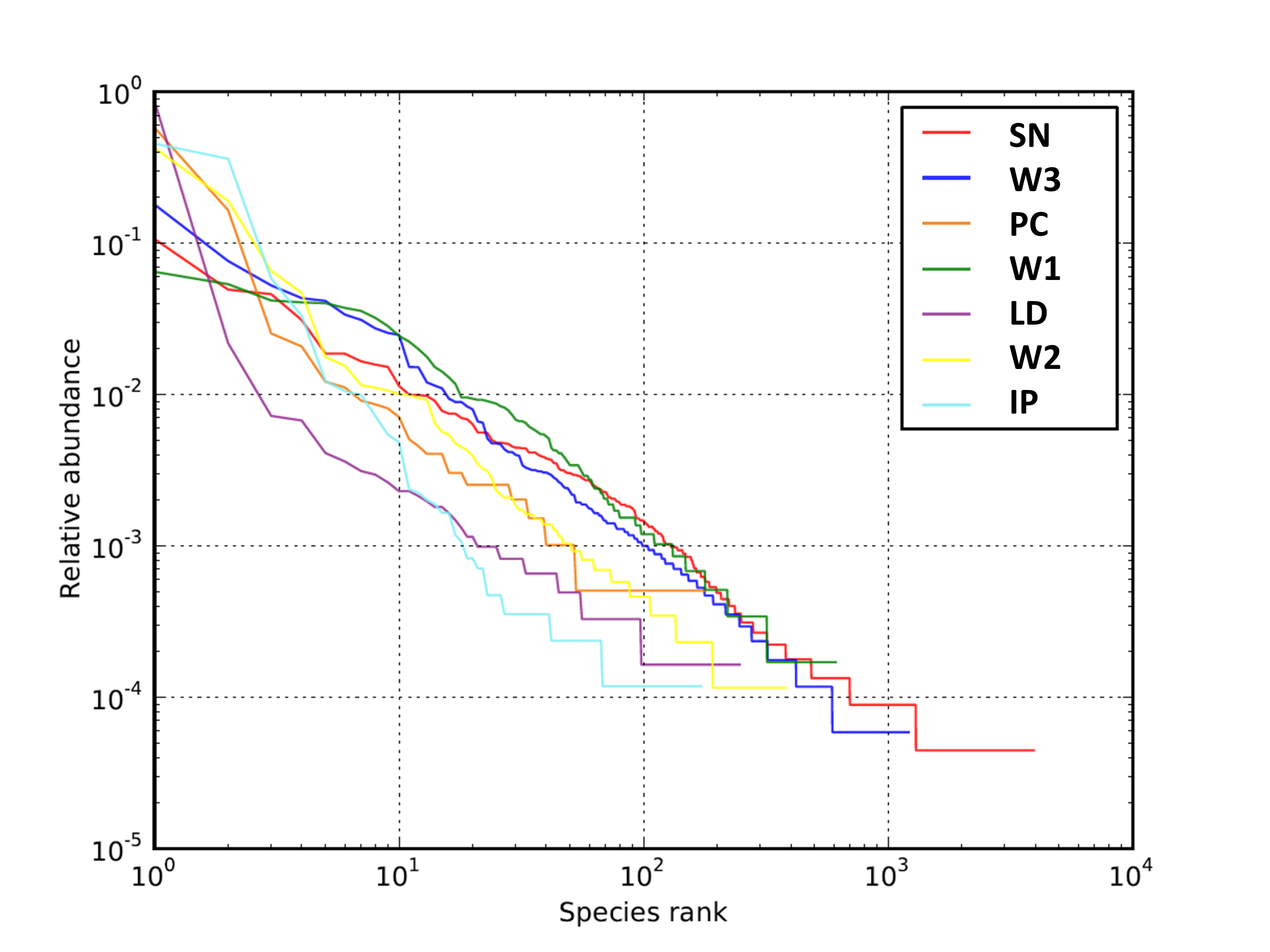

Supplement: Figure S1 — Diversity of microbial communities in deep sea sponges and seawater. Rank abundance curve based on OTUs at 97% similarity. See Table 1 for sample abbreviations. (TIF) [file pone.0091092.s001.tif]

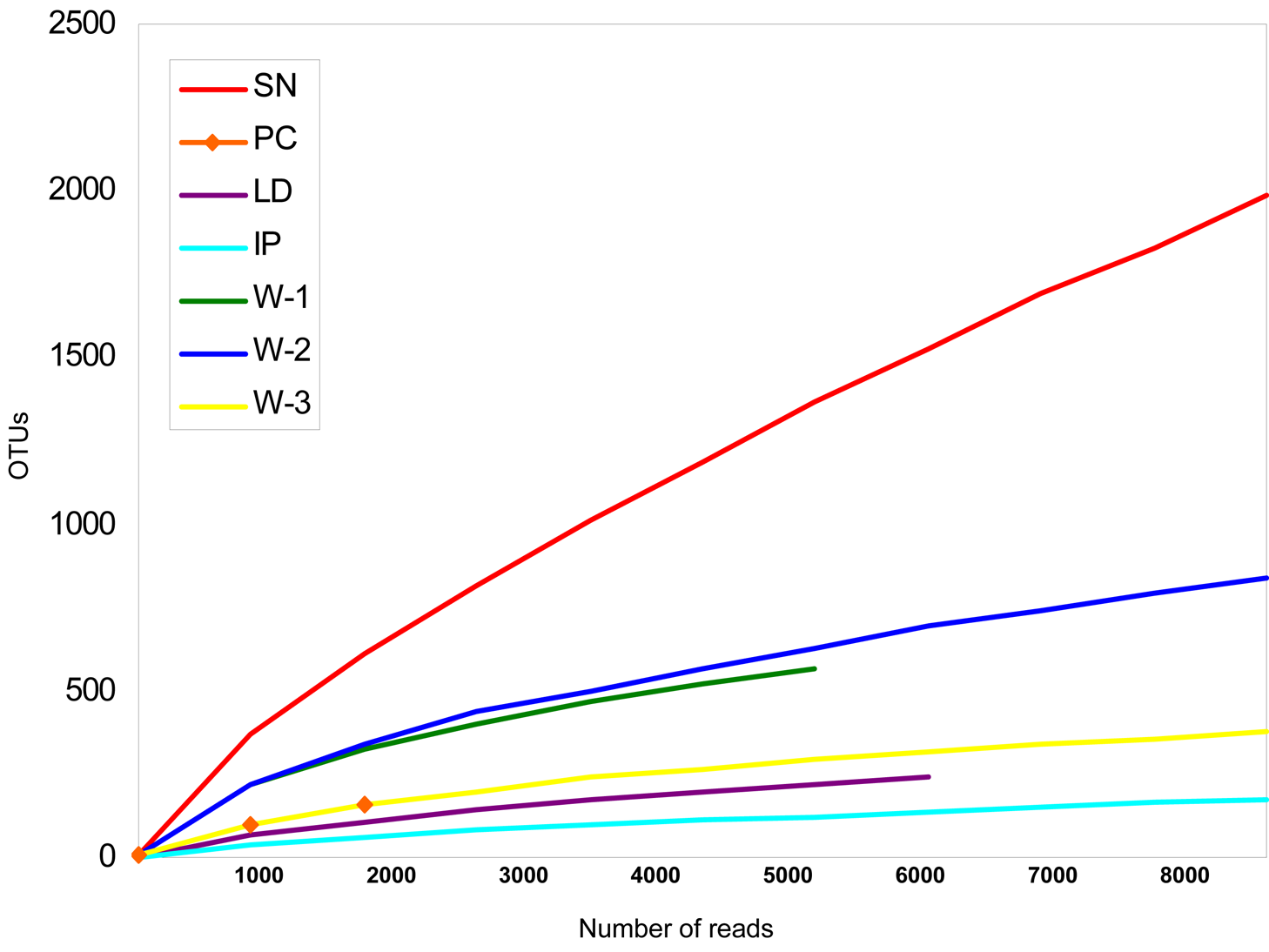

Supplement: Figure S2 — Diversity of microbial communities in deep sea sponges and seawater. Rarefaction curve based on OTUs at 97% similarity. See Table 1 for sample abbreviations. (TIF) [file pone.0091092.s002.tif]
