# Supplementary material for: Evidence of a Putative Deep Sea Specific Microbiome in Marine Sponges
Source: PLoS One. 2014 Mar 26;9(3):e91092. doi: 10.1371/journal.pone.0091092 (PMC3966782; doi:10.1371/journal.pone.0091092)
Supplement: Table S1 — Primer design including Multiplex Identifier (MID). (DOC) [file pone.0091092.s003.doc]

| Primer | Sample | Adapter | Multiplex identifier (MID) | template specific primer |
| --- | --- | --- | --- | --- |
| f | Seawater (W-1) | CGTATCGCCTCCCTCGCGCCATCAG | ACGAGTGCGT | TAGATACCCSSGTAGTCC |
| r |  | CTATGCGCCTTGCCAGCCCGCTCAG | ACGAGTGCGT | CTGACGRCRGCCATGC |
| f | Seawater (W-2) | CGTATCGCCTCCCTCGCGCCATCAG | AGCACTGTAG | TAGATACCCSSGTAGTCC |
| r |  | CTATGCGCCTTGCCAGCCCGCTCAG | AGCACTGTAG | CTGACGRCRGCCATGC |
| f | Seawater (W-3) | CGTATCGCCTCCCTCGCGCCATCAG | AGACGCACTC | TAGATACCCSSGTAGTCC |
| r |  | CTATGCGCCTTGCCAGCCCGCTCAG | AGACGCACTC | CTGACGRCRGCCATGC |
| f | *L. diversichela* | CGTATCGCCTCCCTCGCGCCATCAG | ATATCGCGAG | TAGATACCCSSGTAGTCC |
| r |  | CTATGCGCCTTGCCAGCCCGCTCAG | ATATCGCGAG | CTGACGRCRGCCATGC |
| f | *I. pellicula* | CGTATCGCCTCCCTCGCGCCATCAG | TAGTATCAGC | TAGATACCCSSGTAGTCC |
| r |  | CTATGCGCCTTGCCAGCCCGCTCAG | TAGTATCAGC | CTGACGRCRGCCATGC |
| f | *P. compressa* | CGTATCGCCTCCCTCGCGCCATCAG | ATCAGACACG | TAGATACCCSSGTAGTCC |
| r |  | CTATGCGCCTTGCCAGCCCGCTCAG | ATCAGACACG | CTGACGRCRGCCATGC |
| f | *S. normani* choanosome | CGTATCGCCTCCCTCGCGCCATCAG | CGTGTCTCTA | TAGATACCCSSGTAGTCC |
| r |  | CTATGCGCCTTGCCAGCCCGCTCAG | CGTGTCTCTA | CTGACGRCRGCCATGC |
| f | *S. normani* cortex | CGTATCGCCTCCCTCGCGCCATCAG | CTCGCGTGTC | TAGATACCCSSGTAGTCC |
| r |  | CTATGCGCCTTGCCAGCCCGCTCAG | CTCGCGTGTC | CTGACGRCRGCCATGC |

**Table S1:** Primer design including Multiplex Identifier (MID)
